# A review and recommendations for oral chaperone therapy in adult patients with Fabry disease

**DOI:** 10.1186/s13023-024-03028-w

**Published:** 2024-01-18

**Authors:** Michał Nowicki, Stanisława Bazan-Socha, Beata Błażejewska-Hyżorek, Mariusz M. Kłopotowski, Monika Komar, Mariusz A. Kusztal, Tomasz Liberek, Jolanta Małyszko, Katarzyna Mizia-Stec, Zofia Oko-Sarnowska, Krzysztof Pawlaczyk, Piotr Podolec, Jarosław Sławek, on behalf of the Polish Fabry Disease Collaborative Group

**Affiliations:** 1 Department of Nephrology, Hypertension and Kidney Transplantation, Central University Hospital, Medical University of Lodz, Pomorska 251, 92-213 Lodz, Poland; 2 Department of Internal Medicine, Faculty of Medicine, Jagiellonian University Medical College, Kraków, Poland; 3 2nd Department of Neurology, Institute of Psychiatry and Neurology, Warsaw, Poland; 4 Department of Interventional Cardiology and Angiology, Cardinal Wyszynski National Institute of Cardiology-National Research Institute, Warsaw, Poland; 5 Department of Cardiac and Vascular Diseases, Institute of Cardiology, Jagiellonian University Medical College, Kraków, Poland; 6 Department of Nephrology and Transplantation Medicine, Wroclaw Medical University, Wroclaw, Poland; 7 Department of Nephrology, Transplantology and Internal Medicine, Medical University of Gdańsk, Gdańsk, Poland; 8 Department of Nephrology, Dialysis and Internal Medicine, Medical University of Warsaw, Warsaw, Poland; 9 First Department of Cardiology, Faculty of Medicine in Katowice, Medical University of Silesia, Katowice, Poland; 10 1st Department of Cardiology, Poznan University of Medical Sciences, Poznan, Poland; 11 Department of Nephrology, Transplantology and Internal Medicine, Poznań University of Medical Sciences, Poznan, Poland; 12 Department of Neurological-Psychiatric Nursing, Department of Neurology and Stroke, Faculty of Health Sciences, St. Adalbert Hospital, Medical University of Gdansk, Gdańsk, Poland

**Keywords:** Lysosomal storage diseases, Fabry disease, Migalastat, Oral chaperone therapy

## Abstract

Fabry disease (FD) is a rare, X-linked lysosomal storage disorder affecting both males and females caused by genetic abnormalities in the gene encoding the enzyme α-galactosidase A. FD-affected patients represent a highly variable clinical course with first symptoms already appearing in young age. The disease causes a progressive multiple organ dysfunction affecting mostly the heart, kidneys and nervous system, eventually leading to premature death. Disease-specific management of FD includes enzyme replacement therapy with agalsidase α and β or pharmacological oral chaperone migalastat. Migalastat is a low-molecular-mass iminosugar, that reversibly binds to active site of amenable enzyme variants, stabilizing their molecular structure and improving trafficking to the lysosome. Migalastat was approved in the EU in 2016 and is an effective therapy in the estimated 35–50% of all patients with FD with amenable *GLA* gene variants. This position statement is the first comprehensive review in Central and Eastern Europe of the current role of migalastat in the treatment of FD. The statement provides an overview of the pharmacology of migalastat and summarizes the current evidence from the clinical trial program regarding the safety and efficacy of the drug and its effects on organs typically involved in FD. The position paper also includes a practical guide for clinicians on the optimal selection of patients with FD who will benefit from migalastat treatment, recommendations on the optimal selection of diagnostic tests and the use of tools to identify patients with amenable *GLA* mutations. Areas for future migalastat clinical research have also been identified.

## Introduction

Fabry disease (FD) (Anderson–Fabry disease, Online Mendelian Inheritance in Man [OMIM] number 301500, ORPHA:324) is a rare, X-linked lysosomal disorder caused by genetic abnormalities in *GLA*, the gene encoding the enzyme α-galactosidase A (α-Gal A) [1, 2]. The prevalence of the disease has been estimated at one in 40,000–117,000 worldwide, but some newborn screening estimate prevalence of 10 times higher [3]. The decreased activity of α-Gal A leads to progressive, pathological accumulation of glycolipids, such as globotriaosylceramide in lysosomes, leading to lysosomal dysfunction and cell degeneration, followed by tissue inflammation and organ fibrosis. The disease affects males and females and may present as “classical”, “later-onset” or “non-classical” forms. Clinical signs in the classic form appear in males during childhood or early adolescence but usually later in females. The most typical early symptoms are neuropathic pain (acroparesthesia), including “pain crisis”, gastrointestinal symptoms, such as abdominal pain and vomiting, heat and cold intolerance, and sweating abnormalities. These eventually progress to organ dysfunction including chronic kidney disease with sub-nephrotic proteinuria, hypertrophic cardiomyopathy associated with cardiac arrhythmia, and recurrent cardio- or cerebrovascular events. The life expectancy of affected patients is significantly reduced. Less common manifestations of FD consist of ocular (cornea verticillata) and neurological (hearing impairment, vertigo, and tinnitus) abnormalities, hypohidrosis, and skin changes (angiokeratomas). Patients with non-classical or late-onset FD show delayed manifestations or a single-organ involvement, e.g., hypertrophic cardiomyopathy, and usually a better clinical prognosis. The diagnosis of Fabry disease is frequently hindered by many years due to the non-specific and complex nature of the presenting signs and symptoms. In male patients, FD diagnosis involves demonstrating α-Gal A deficiency in leucocytes. In females, the diagnostic gold standard consists of molecular genetic testing due to the potentially high residual enzymatic activity. However, at diagnosis, *GLA* sequencing and measurement of plasma globotriaosylsphingosine (lyso-Gb3), a renewed FD circulating biomarker, is recommended in both genders. Recognition of FD is essential as effective treatments are now available and early therapy initiation is critical to improving the major affected organs from irreversible damage [1, 2].

Management of FD includes FD-specific therapy and supportive care for gastrointestinal symptoms, pain, and renal and cardiac function. Currently, available FD-specific treatment includes enzyme replacement therapies (ERTs) with recombinant α-Gal A (agalsidase α and agalsidase β) and pharmacological chaperone oral therapy with migalastat [2]. The National Health Fund in Poland reimburses all three drugs in the therapeutic program No: B104 [4]. In addition, novel ERT formulations, gene therapy, and substrate reduction therapy are in development. Among them in clinical or preclinical trials are pegunigalsidase-alfa (Protalix Biotherapeutics, Israel) and moss-aGal (Greenovation biopharmaceuticals, Germany), plant-origin ERT medications, as well venglustat (Sanofi Genzyme) and lucerastat (Idorsia Pharmaceuticals, Switzerland), molecules used for substrate reduction [5]. The clinical effects and safety of these candidate therapies need to be confirmed in ongoing clinical trials.

Previously, our expert group provided detailed characteristics of enzyme replacement therapy option, including data on its efficacy, contraindications, side effects, and principles of its initiation and discontinuation in FD patients in Poland [2]. The current statement and recommendations refer to the oral chaperone migalastat.

## Migalastat—drug characteristics and its pharmacological properties

Migalastat, under the brand name of Galafold, is manufactured by Amicus Therapeutics and was approved by the European Medicines Agency (EMA) in May 2016 as the first oral drug for FD [6]. It is a low-molecular-mass iminosugar, 1-deoxygalactonojirimycin, formulated as migalastat hydrochloride 150 mg. It is an analog of the terminal galactose of globotriaosylceramide (GL-3), an α-Gal A substrate. It binds reversibly to the active site of susceptible enzyme variants, stabilizing their molecular structure and improving transport to the lysosomes. After then, the drug dissociates due to the acidic pH in lysosomes, and the lysosomal α-Gal A concentration increases. However, for an effective chaperone therapy, patients must have amenable *GLA* variants as determined by the GLP HEK assay (described below), representing an estimated 35–50% of all known mutations [7–9]. Therefore, their precise identification is of critical significance, particularly since many *GLA* variants are new “private” mutations [7, 10]. The majority of amenable mutations, especially missense ones, are related to the misfolded α-Gal A protein, which cannot properly enter the lysosomes, leading to premature enzyme degradation in the endoplasmic reticulum [8, 11]. On the other hand, large mutations, such as frameshift or splicing mutations, are classified as non-amenable since they do not produce any enzyme; and do not require further evaluation [8].

## Identification of amenable alpha-galactosidase A gene variants

The identification of amenable mutation is based on in vitro studies, with the Good Laboratory Practice-Human Embryonic Kidney (GLP-HEK) assay developed by Benjamin et al. [10] as a gold standard. The assay uses HEK-293 cells transfected with wild-type *GLA* (normal α-Gal A activity) as control and mutant *GLA* to measure α-Gal A in the presence or absence of migalastat. Patients are eligible for treatment with migalastat if the amended α-Gal A activity rises at least 1.2-fold relative to baseline and with an absolute increase in enzymatic activity of at least 3% relative to wild-type *GLA* [10]. The large variability of in vitro biochemical response to migalastat, i.e., 1.2–30.4-fold changes, depending on *GLA* variant, may explain the extensive range of α-Gal A activity change in vivo after the migalastat therapy [10]. However, in vitro GLP HEK assay enzyme activity increases do not correlate directly with individual patient response. Furthermore, the degree of enzyme activity increase has not been found to correlate with long term renal outcomes [7, 8, 12, 13].

Thus, clinical monitoring, discussions about adherence, and consecutive measurements of the α-Gal A enzymatic activity in leukocytes and plasma lyso-Gb3 levels are critical in all migalastat-treated patients, particularly at the beginning [14]. Furthermore, as different laboratories have developed specific assays to investigate lysosomal α-Gal A activity, conclusions must be taken cautiously [8, 15, 16].

For a newly diagnosed patient, we recommend using https://www.galafoldamenabilitytable.com/ which lists all tested amenable and non-amenable mutations. This is the only clinically validated tool to assess amenability to migalastat based on the GLP HEK assay.

## Pharmacokinetics and pharmacodynamics of migalastat

The recommended dose of migalastat for all treated subjects is 123 mg, taken once every other day at the same time of day. The dose is based on its pharmacodynamic and pharmacokinetic properties. As food decreases migalastat absorption by approximately 40%, the medication needs to be taken at least 2 h before and 2 h after a meal and must be consumed with clear liquid. In addition, caffeine should not be consumed during this 4 h fasting window. The capsule must be swallowed whole; its cutting, crushing, or chewing is forbidden. If a daily dose is missed, the patient should take the forgotten capsule if less than 12 h from the scheduled time have passed. After there, the alternate-day dosing schedule needs to be adjusted [6].

The pharmacokinetics of migalastat is not dependent on gender or age over 12 years. Following oral administration, the absolute bioavailability of migalastat is estimated to be 75% [17, 18]. Migalastat is rapidly absorbed, reaching peak plasma concentrations around 3 h after a single oral dose. The drug is extensively distributed into tissues, with no detectable plasma protein binding and a half-life of 4 h. Pharmacokinetic studies revealed that approximately 77% of the migalastat dose is excreted in the urine, predominantly unchanged. The drug does not inhibit or induce cytochrome P450 enzymes and is unlikely to interfere with major human transporter proteins.

No direct studies have been carried out in subjects with impaired hepatic function; however, from the excretion and metabolic pathways, it is unlikely that liver disease may significantly affect the pharmacokinetics of migalastat. Migalastat has also not been studied in FD patients with an estimated glomerular filtration rate (GFR) of less than 30 ml/min/1.73 m^2^, thus should not be initiated in that group of patients [6]. It is unclear whether migalastat may cross the blood–brain barrier in humans. Still, in a mice FD model, migalastat increased α-Gal A activity and reduced GL-3 levels in most tissues, including the brain [19].

In another study in transgenic mice, oral administration of migalastat reduced elevated lyso-Gb3 levels up to 64%, 59%, and 81% in the kidney, heart, and skin, respectively, equal to or greater than observed for GL-3. In the same study, oral administration of migalastat HCl (150 mg QOD) reduced urine GL-3 and plasma lyso-Gb3 in three of six patients enrolled in phase 2 studies [20].

## The safety and tolerability of migalastat

The migalastat tolerance was investigated in two phase 3 randomized clinical trials (RCTs): FACETS [21, 22] and ATTRACT [9, 23]. The data on adverse events (AEs) has also been collected in several prospective observational studies [13, 24, 25]. Therapy with migalastat, in general, was safe and well tolerated. In clinical trials, investigators reported severe adverse events (SAEs) in 2 patients during the first 6 months of therapy and in 4 patients in the placebo group; 5 cases in stage 2 (months 6–12), including 2 incidents of pulmonary embolism and deep vein thrombosis. Furthermore, 11 AEs were documented in the open-label extension phase [7, 8]; however, only 2 of them, i.e., fatigue and paresthesia demonstrated in the same patient, were reported as likely migalastat-related. In addition, despite the relatively frequent occurrence of mild and moderate AEs (10–100%), no treatment discontinuation was required [9, 13, 21–25].

The most frequently reported adverse event that occurred during migalastat treatment was headache in both the classic phenotype (4/14 [29%]) and other patients (15/36 [42%]) subgroups. The later subgroup did not report any other adverse events, whereas diarrhea, procedure pain, and vertigo were reported in ≥ 20% of patients in the classic phenotype subgroup [22].

Migalastat had no impact on clinical routine laboratory tests (chemistry, hematology, and urinalysis) or on electrocardiograms [23]. In an animal model, however, using much higher doses than in humans, a reversible anti-spermatogenic effect was observed [26].

## Therapeutic efficacy of migalastat in Fabry disease depending on the organ invoαed

The clinical effectiveness of migalastat in FD patients has been evaluated in two phase 3 clinical trials, i.e., FACETS [21] and ATTRACT [9], and in some real-world observations. FACETS study comprised a 6-month randomized, double-blind, placebo-controlled phase, followed by a 6-month open-label extension (OLE) phase with the crossover of patients in the placebo arm to receive migalastat 123 mg every other day (equal to 150 mg migalastat hydrogen chloride), and a 12-month migalastat treatment extension phase. It included 50 FD naïve patients with the amenable mutation. Male to female ratio was 44:56%, and 94% had a multiorgan disease. The ATTRACT study was an open-label, randomized study comprising an 18-month active-controlled (ERT), randomized phase, followed by a 12-month optional OLE phase with a crossover of patients in the ERT arm to receive migalastat 123 mg every other day. It consisted of 57 patients aged 16–74 years (56% females; 89% with multiorgan disease) receiving ERT before study enrolment, randomized into migalastat or ERT continuation (ratio: 1.5:1) [27].

## Cardiovascular effects

Both RCTs have demonstrated that migalastat might affect cardiac FD endpoints. In the FACETS study [21], migalastat use in 44 patients with amenable mutations was associated with a significant decrease in the left ventricular mass index (LVMi) (− 7.7 ± 3.7 g/m^2^), particularly in those with left ventricular hypertrophy (LVH) at baseline (− 18.6 ± 8.3 g/m^2^, n = 11). In addition, the mean end-diastolic interventricular septum thickness changed by − 0.06 ± 0.05 cm from the baseline mean value of 1.17 ± 0.06 cm, representing a mean decrease of 5.2%. The end-diastolic left ventricular posterior wall thickness remained stable for up to 24 months [21]. Interestingly, a recent post hoc analysis of the FACETS trial [22] revealed that migalastat might be even more beneficial in the subset of male patients with the classic phenotype regarding cardiac hypertrophy. The authors compared the patients with the classical phenotype, migalastat-amenable variants, and residual peripheral blood mononuclear cell α-Gal A activity < 3% to the remaining male patients who did not meet classic phenotype criteria and all females. They showed that migalastat led to reductions in LVMi in both subgroups. Notably, baseline LVMi values were 114.3 ± 27.3 g/m^2^ in males with the classical phenotype (n = 7 [50%] with documented hypertrophic cardiomyopathy) and 88.2 ± 32.3 g/m^2^ in the remaining patients (n = 4 [11%] with documented hypertrophic cardiomyopathy). The mean LVMi change from baseline to month 24 was − 16.7 ± 18.64 g/m^2^ in the former (n = 9) and − 3.2 ± 18.66 g/m^2^ in the later (n = 18) groups.

The ATTRACT trial [9, 27] also showed that migalastat significantly reduced LVMi compared with ERT. LVMi was decreased considerably at month 18 from baseline (mean and 95% confidence interval [CI] − 6.6 [− 11.0 to − 2.2] g/m^2^) in migalastat-treated patients but not in those who continued ERT (mean and 95% CI − 2.0 [− 11.0 to 7.0] g/m^2^). The most extensive LVMi changes on migalastat were observed in those presenting LVH at baseline (9 females and 4 males; mean LVMi 116.7 g/m^2^; the change from baseline to month 18, mean and 95% CI − 8.4 [− 15.7 to 2.6] g/m^2^) [9, 27]. Additionally, a subsequent 12-month, migalastat-only, open-label extension of the ATTRACT study [23] (30 months of therapy in total) revealed stable LVMi parameters in the majority and a significant decrease in those with LVH at baseline (mean and 95% CI − 10.0 [16.6 to − 3.3] g/m^2^).

The RCT data are consistent with the results of real-life observations. For example, the prospective multicenter FAMOUS study [13] assessed for 24 months the safety and efficacy of migalastat with a particular focus on LVMi (primary endpoint) and renal function (secondary endpoint) in 60 patients both previously treated with ERT or ERT-naïve. Mean LVMi decreased significantly by 10.2 (95% CI 5.3–15.2) g/m^2^ (108.6 ± 48.0 vs. 98.4 ± 41.4 g/m^2^, p = 0.001) and was documented in both males and females.

Also, in another German single-center study including 14 patients (11 males, 3 females), 12 months of migalastat therapy led to a myocardial mass decrease from 137 (IQR 86–159) to 130 (IQR 82–169) g/m^2^ (p = 0.012) [24].

The results of the single-center observational study evaluating the impact of the ERT switch to migalastat [22] on cardiovascular measures are also worth noting. In 7 naïve males, heart involvement was analyzed at baseline, after 12 months of ERT, and after a further year of migalastat therapy. In patients who had normal systolic and diastolic parameters at baseline, they remained stable for the whole period. However, for those with baseline LVH, a significant decrease of LVMi was reported after ERT (p = 0.028) and also after subsequent treatment with migalastat for one year (p = 0.016), both vs. baseline [25].

However, since all presented cardiac data were evaluated in 2D transthoracic echocardiography, we should be aware of the procedure limitations. Both inter- and intra-observer variability, the intrinsic accuracy, and the precision of the measurements could make some results, particularly in small groups, uncertain [6].

Recently, however, the results of the Migalastat on cArdiac Involvement in FabRry Disease (MAIORA) study were published, designed to assess migalastat’s effect on FD cardiac involvement, combining left ventricular morphology and tissue characterization by cardiac magnetic resonance imaging (MRI) with cardiopulmonary exercise testing [28]. The study included 16 treatment-naïve FD patients (4 women) with cardiac involvement. An 18-month treatment with migalastat stabilized the left ventricular mass, and there was a trend toward improving exercise tolerance and septal T1 in MRI increase.

## Renal effects

The primary objective of the FACETS [21] study was to compare the effect of migalastat vs. placebo on renal GL-3 deposits, expressed as the number of inclusions in kidney interstitial capillaries. Kidney biopsies were conducted at baseline and after 6 and 12 months of the treatment. The other study objectives were the comparisons of migalastat vs. placebo on renal function, 24-h urinary protein excretion, and urinary GL-3 levels. At 6 months, in 25 amenable patients who received migalastat therapy, a significant reduction in the GL-3 inclusions per kidney interstitial capillary was seen, which remained stable at the 12 months. Similarly, a substantial decrease in kidney GL-3 was observed after 6 months of migalastat in those who switched after 6 first placebo months. In addition, the mean total GL-3 inclusion volume per podocyte decreased after 6 months on migalastat [21, 29]. That is an important finding since podocyte clearance from GL-3 is challenging.

From a clinical point of view, 6 months of migalastat was associated with stable proteinuria and 24-h urinary GL-3 excretion and estimated or measured glomerular filtration rate (GFR) compared to placebo. In the follow-up study, in patients treated with migalastat for up to 24 months, the mean yearly changes of GFR from baseline in both estimated and directly measured (iohexol clearance) methods were low and evaluated as − 0.30 ± 0.66 and − 1.51 ± 1.33 ml/min/1.73 m^2^, respectively. As expected, the estimated (e)GFR reduction was more remarkable in males and those with higher proteinuria at baseline.

In ATTRACT study [9], no significant differences were observed in GFR decline between the migalastat group and those who continued ERT. Mean annualized GFR loss was − 0.40 ± 0.93 and − 1.03 ± 1.29 ml/min/1.73 m^2^/year for eGFR (Chronic Kidney Disease—Improved Prediction Equations [CKD-EPI]) and − 4.35 ± 1.64 and − 3.24 ± 2.27 ml/min/1.73m^2^/year for directly measured, in migalastat and ERT group, respectively. Interestingly, the mean changes from baseline in 24-h urine protein excretion were numerically lower in the migalastat group (49.2 mg) than in the ERT group (194.5 mg).

The results of the long-term follow-up of migalastat therapy in patients participating in both FACETS and ATTRACT, and in the open-label extensions phases were summarized by Bichet et al. [12]. Altogether, 78 patients completed the observational phase with a median treatment duration of 7.0 years in ERT naïve patients and 5.1 years in those on ERT. The annualized overall loss of GFR was − 1.6 (3.1) Ml/min/1.73 m^2^ and − 1.6 (3.6) ml/min/1.73 m^2^, in ERT naïve and ERT-experienced patients, respectively. As expected, GFR loss was more significant in males than females and higher in patients with classic phenotypes vs. others. Furthermore, Feldt-Rasmussen et al. [23] showed long-term renal function stability after 30 months of migalastat therapy in an open-label extension phase.

Another long-term, a real-life German study reported a moderate eGFR loss on migalastat in 54 patients (26 women). After 24 months of treatment, an annual eGFR loss was − 5.3 ± 10.8 Ml/min/1.73 m^2^ and − 8.9 ± 14.1 ml/min/1.73 m^2^ in females and males, respectively [30]. At 24 months, multivariable regression analyses showed that antihypertensive treatment may be associated with a more prominent loss of eGFR (P = 0.0834). The patients receiving angiotensin-converting-enzyme (ACE) inhibitors, angiotensin II receptor blockers (ARBs) or aldosterone inhibitors had a significant decrease in eGFR from baseline (p < 0.0001 and p = 0.0003 at 12- and 24-months’ follow-up, respectively) and those receiving migalastat with ‘other’ or ‘no’ antihypertensive treatment had stable eGFR from baseline [30]. In an earlier single-center study from Germany, 12 months of migalastat treatment of 14 both naïve and previously ERT-receiving patients was related to a significant increase in α-Gal A activity and a reduction in lyso-Gb3 [24]. Nevertheless, eGFR CKD-EPI decreased from 87 (interquartile range [IQR]: 75.5–102) to 78 (IQR: 71.5–99) ml/min/1.73m^2^. In another real-life study, Riccio et al. [25] showed that eGFR remained unchanged and proteinuria decreased on migalastat.

## Neurological effects

Clinical trials with migalastat and long-term follow-up studies did not permit separate analysis of neurological events as part of the primary endpoint due to study group size limitation [9, 21, 22, 27, 29] with the exception of pain [31, 32]. However, in Germain et al. study [21], neurological symptoms were already present at baseline as 3 of 14 patients had a history of TIA or stroke, 3 reported acroparesthesias, and 6 were already diagnosed with hearing loss. However, the changes in neurological status were not studied, and cerebrovascular events were not reported. In the study of Riccio et al. [25] 7 male Fabry patients were switched from ERT to migalastat and observed for 1 year. Neurologic changes were determined on the basis of clinical examination, interviews regarding vascular episodes and the MRI results. No patient experienced cerebrovascular events during both the prior ERT (12 months) and during subsequent 12-month migalastat therapy. The pain symptoms and their intensity also did not change during the study. In contrast, a recently published prospective multicenter ‘MigALastat Therapy Adherence among FABRY patients’ (MALTA-FABRY) study, which used the dedicated Fabry Pain Questionnaire (FPQ) showed a mild reduction in pain encompassing both its frequency and intensity in 23 migalastat-treated patients in less than 12 months after initiation of the therapy [31].

Neurological symptoms, primarily headaches and vertigo, were reported as adverse events in a majority of migalastat studies (25–42%, and 20%, respectively) [21, 22]. However, in the phase 3 ATTRACT study, the low proportion of patients with cerebrovascular events (one in the ERT group and none of those receiving migalastat) did not allow for firm conclusions [27]. Further studies involving a larger population of FD patients with cerebrovascular disease and/or white matter lesions are needed to assess the drug's potential role in limiting the progression of neuronal damage.

## Fabry-associated composite clinical events, patient-reported quality of life and long-term adherence to therapy

Recently a post hoc analysis of four phase 3 RCTs and their extension phases has been published. This study evaluated the incidence of composite outcomes, including renal, cardiac, and cerebrovascular events (Fabry-associated clinical events—FACEs) in 97 patients without or with a history of ERT treatment who were treated with migalastat for up to 8.6 years (median: 5 years) [32]. In the study group, 17 patients (17.5%) experienced 22 FACEs but no deaths. The incidence rate of FACEs was 48.3 per 1000 patient-years. Numerically higher incidence rates were observed in men versus women and in patients older than 40 years.

It should also be noted that in this post-hoc analysis, renal impairment at baseline was the most significant risk factor for the earlier occurrence of the composite (renal, cardiac or cerebrovascular) events during long-term treatment with migalastat [32]. Furthermore, life quality and long-term adherence to oral therapy with migalastat were evaluated in 40 patients (19 women) as a part of the Prospective multicenter ‘MigALastat Therapy Adherence among FABRY patients’ (MALTAFABRY) study [31]. Outcome measurements were reported over a follow-up period of 24 months. More than 90% of patients (37 of 40) adhered well to therapy, independent of gender. Also, FD patients reported a significant improvement in pain, better physical activity tolerance, and stable quality of life with an improvement in SF-36 score between baseline and 24-month follow-up (average change from baseline: 8.57 [95% CI 1.32–15.8] points, p = 0.022, n = 28).

## Selection of patients for migalastat treatment

Migalastat is recommended as a therapeutic option only for FD patients with amenable *GLA* variants. Qualification for migalastat depends on age, sex, symptoms, and the stage of the disease in affected organs [6, 21, 33]. The inclusion, exclusion criteria, and contraindication for migalastat therapy are summarized in the table below (Table 1).

**Table 1:** Inclusion criteria, contraindications for migalastat treatment, and exclusions from the therapy

Inclusion criteria
Age ≥ 12 years old
Symptomatic classic phenotype or late-onset Fabry disease (at least one organ affected)
Sex-specific criteria:
Men: significant deficiency of α-galactosidase A in a dried blood test, plasma, leukocytes, fibroblasts
The presence of a pathogenic amenable mutation
Females—deficiency of α-galactosidase A (in case of symptoms is not required)
The presence of a pathogenic amenable mutation
GFR ≥ 30 ml/min/1.73 m^2^
Contraindications
Age < 12 years old
Non-amenable genetic variants
Simultaneous ERT
Dialysis therapy
GFR < 30 ml/min/1.73 m^2^
Pregnancy and lactation
Hypersensitivity reaction to the drug or excipient
Asymptomatic form of the disease
Heart: advanced stage of the disease with diffuse myocardial fibrosis, NYHA IV, no possibility of heart transplantation if the heart is the only organ involved
CNS: advanced changes
Kidney: end-stage failure without the possibility of kidney transplantation
End-stage of Fabry disease or existing comorbidities or another congenital malformation with an unfavorable prognosis
Exclusions from the therapy
Hypersensitivity reaction
Serious adverse events
Pregnancy and lactation
No improvement despite treatment
Lack of cooperation with the patient

### Migalastat dosage

As mentioned above, migalastat is recommended in an oral 123 mg dosage given once every other day at the same time.

Dosage modification in patients with renal function impairment:

- Mild to moderate (eGFR ≥ 30 ml/min/1.73 m^2^): no dosage adjustment,
- Severe (eGFR < 30 ml/min/1.73 m^2^): drug is not recommended,
- After a kidney transplant, if satisfactory organ function is, the drug might be prescribed in regular dosage.

### Therapy monitoring

Migalastat therapy is safe and generally well-tolerated. Headache and nasopharyngitis are the most frequent side effects, usually mild to moderate and unrelated to therapy cessation.

Migalastat therapy requires clinical and biochemical monitoring. The FAMOUS study [13] showed that some treated patients might develop low blood pressure. Therefore, close blood pressure monitoring based on 24-h home blood pressure measurements is recommended for all patients with FD, regardless of the type of therapy, to avoid values below 120 mmHg.

The patients on migalastat in Poland undergo obligatory evaluations every 6 months as a part of the treatment program, including consults with their primary treating physician, nephrologist, cardiologist, and neurologist. Each assessment includes a 12-lead ECG, 24-h ECG, transthoracic echocardiogram, blood biochemistry with eGFR calculation, and urinalysis to evaluate proteinuria and albuminuria. The patients are also examined for the severity of FD symptoms (e.g., neuropathic pain and gastrointestinal symptoms) and complete a quality-of-life questionnaire. Treatment tolerance and side effects are also essential parts of the evaluation.

During the treatment with migalastat, our expert group advises assessing cardiac biomarkers, i.e., high sensitivity cardiac troponin and NT-pro-BNP, as well as leukocyte α-Gal A activity, serum lyso-Gb3, and urine GL-3.

In stable disease, we suggest heart and brain MRI in eligible patients every 3 years, or more frequently if clinically indicated.

Monitoring of migalastat treatment is recommended in some European countries, Canada, and Australia [6, 34]. In case of significant clinical deterioration, alternative therapy should be considered.

Migalastat therapy is not allowed during pregnancy or breastfeeding [6]; thus, an effective contraceptive method or sexual abstinence is required by treated women. Before beginning treatment, a urine pregnancy test needs to be performed. The long-term registry data of pregnant and/or breastfeeding women exposed to at least one dose of migalastat are being collected as a part of US Food and Drug Administration program [35].

The patient’s adherence is assessed during each follow-up visit and before dispensing the next refill of medication. Furthermore, we suggest a monthly telemedicine visit in the first 3 months of therapy to check for side effects and adherence.

In case of a severe adverse event or a sudden clinical deterioration, the patient must report this to the treating physician to decide whether the treatment may be continued.

During the 6-monthly follow-up visits, the therapeutic goal of FD therapy should be an improvement or stabilization of FD symptoms, including quality of life. [13, 33, 36]. Since plasma lyso-Gb3 may not be a suitable biomarker for monitoring treatment response to migalastat [35] we recommend that a lack of lyso-Gb3 response should not be interpreted as a failure to meet the therapeutic target unless accompanied by a deterioration of symptoms and/or function of at least one typically-affected organ.

### Polish experience with migalastat therapy

At the end of December 2023, 32 FD patients are treated with migalastat in Poland, and none was discontinued. In June 2022, we conducted a multicenter questionnaire survey (unpublished data) with a methodology similar to the GALA project [37]. Each questionnaire item was presented to define the degree of agreement on a precise statement, according to a 3-point Likert scale (1 = disagrees, 2 = neither agrees or disagrees, 3 = agrees). The responses to the questionnaire were collected by an online system and analyzed. At that moment, 15 individuals were treated with migalastat, with an average therapy duration of 10 months and the longest period of 2 years. The therapy was initiated in 10 treatment naïve patients and in 5 switched from ERT.

The results of that survey is presented in Table 2. Among expert physicians (Polish FD collaborating group) from different backgrounds (cardiology, nephrology, neurology), an agreement was achieved that in patients (both genders) aged ≥ 12 years with amenable mutations and classic FD (plus in males with non-classic FD), migalastat therapy is recommended in the presence of symptoms or signs of organ damage. Complete agreement was recorded to use migalastat in the following conditions: rhythm disorders, renal involvement (albuminuria, proteinuria, eGFR 90–30 ml/min/1.73 m^2^), gastrointestinal symptoms, and severe mobility impairment.

**Table 2:** Results of the questionnaire assessing the Polish experts’ opinion regarding migalastat use in Fabry disease (FD); the level of agreement is presented as percentage of physicians (*n* = 5) prescribing migalastat

Question	Disagreement (disagree + strongly disagree)	Neither agreement or disagreement	Agreement (agree + strongly agree)
When compared with i.v., oral therapy can improve QoL in patients with FD	0	0	100
According to available data, migalastat can be considered a safe and effective treatment for FD	0	0	100
According to available evidence, one of the advantages of migalastat over ERT is its superior efficacy on heart damage	0	60	40
Poor compliance to oral therapy with migalastat can be an issue	20	60	20
Migalastat therapy can be taken into consideration as an alternative to ERT in patients with FD and amenable mutations	0	0	100
In a male patient aged ≥ 12 years with an amenable mutation and classic FD, migalastat therapy might be taken into consideration at diagnosis, even when signs/symptoms of organ damage are lacking	40	20	40
In a male or female patient aged ≥ 12 years with an amenable mutation and classic FD, migalastat therapy is recommended in the presence of signs/symptoms of organ damage	0	0	100
In a male patient aged ≥ 12 years with an amenable mutation and non-classic FD, migalastat therapy is recommended in the presence of signs/symptoms of organ damage	0	0	100
In a female patient aged ≥ 12 years with an amenable mutation and non-classic FD, migalastat therapy might be taken into consideration at diagnosis, even when signs/symptoms of organ damage are lacking	40	40	20
In a female patient aged ≥ 12 years with an amenable mutation and non-classic FD, migalastat therapy might be considered at the first onset of signs/symptoms of organ damage	20	0	80
Migalastat therapy is recommended in a patient with FD aged ≥ 12 years, with an amenable mutation and cardiac hypertrophy (≥ 11 mm)	20	0	80
Migalastat treatment should be considerated in a patient aged ≥ 12 years with FD, with an amenable mutation and rhythm disorders (sinus bradycardia, atrial fibrillation, extrasystole) and/or electrocardiogram alterations	0	0	100
Migalastat therapy is recommended in a patient with FD aged ≥ 12 years with an amenable mutation and pathological microalbuminuria (according to KDIGO guidelines)	0	0	100
Migalastat therapy is recommended in a patient with FD aged ≥ 12 years with an amenable mutation and proteinuria (according to KDIGO guidelines)	0	0	100
Migalastat therapy is recommended in patients aged ≥ 12 years with FD, amenable mutations and eGFR 60–90 ml/min/1.73 m^2^ (CKD-EPI) with evidence of renal function decline progression (> − 1 ml/min/1.73 m^2^/year)	0	0	100
Migalastat therapy is recommended in patients aged ≥ 12 years with FD, amenable mutations and eGFR 30–60 ml/min/1.73 m^2^ (CKD-EPI)	0	0	100
Migalastat treatment may be considered in patients aged ≥ 12 years with FD, amenable mutations and progression of white matter lesions	0	40	60
Migalastat treatment may be considered in patients aged ≥ 12 years with FD, amenable mutations and history of TIA/stroke	0	40	60
Migalastat treatment may be considered in patients aged ≥ 12 years with FD, amenable mutations and progressive loss of hearing (corrected by age)	0	40	60
Migalastat treatment should be considered in patients aged ≥ 12 years with FD, amenable mutations and gastrointestinal symptoms	0	0	100
Migalastat treatment should be considerated in patients aged ≥ 12 years with FD, amenable mutations and acroparesthesia, even if controlled by symptomatic therapy	0	20	80
In a patient aged ≥ 12 years with an amenable mutation, currently on ERT, switching to migalastat should be considered in unstable disease and/or poor therapy response	0	20	80
In a patient aged ≥ 12 years with an amenable mutation, currently on ERT, switching to migalastat should be considered in uncontrolled infusion reactions and/or poor compliance	0	20	80
In a patient aged ≥ 12 years with an amenable mutation, currently on ERT, switching to migalastat should be considered in movement/mobility impairment affecting difficulties in transport for ERT infusions	0	0	100

*CKD-EPI* Chronic kidney disease-improved prediction equations, *eGFR*-estimated glomerular filtration rate, *ERT* Enzyme replacement therapy, *FD* Fabry disease, *KDIGO* Kidney disease improving global outcomes, *TIA* Transient ischaemic attack

The available local Polish data consistently suggest that migalastat is a reasonable FD therapeutic option, even though data from longer-term follow-ups are still coming.

## Indications for Fabry disease-specific treatment change

Available data showed that FD patients with amenable mutations on long-term ERT switched to migalastat showed stability in cardiac parameters and renal function [9]. Therefore, an oral chaperone is likely not inferior to ERT and would be preferable in treating those who have amenable mutations and want to avoid biweekly venipuncture, such as pediatric patients. However, both migalastat phase 3 RCTs [9, 21] involved mainly patients with the milder disease (LVMi, mean ± SD: 93.9 ± 29.6 g/m^2^; eGFR, mean ± SD: 91.2 ± 22.5 ml/min/1.73 m^2^; urine 24-h proteinuria, median [0.25–0.75 quartile range]: 198 [0–5566 mg]) [38]. Thus, data on the migalastat efficacy in more advanced diseases, such as chronic kidney disease stage 3, nephrotic proteinuria, and more extensive LVH are limited. Likewise, this relatively new medication’s long-term effectiveness and safety are unknown, although both seem beneficial [12, 32]. Nevertheless, some on ERT, even with advanced disease form, may prefer to switch to migalastat with the possible issues of difficult intravenous access, anti-drug antibodies, and infusion-associated adverse reactions. Notably, switching from ERT (where a medical staff supervises the infusions) to migalastat (where the patient must remember to take the pills every other day) may be related to noncompliance, particularly in those with memory deficits. Therefore, the patients need to be carefully monitored in case of switching from ERT to migalastat, including clinical symptoms, discussions with the patient about adherence, and cardiac and kidney function, assessed at least every 6 months during the first 3–5 years after changing.

In patients with more severe disease but with eGFR above 30 ml/min/1.73 m^2^ and with an amenable mutation, some recommendations (e.g., Canadian) [39] suggest newly starting disease-specific therapy, in addition to considering treatment with ERT or migalastat, also a third option, i.e., starting with ERT and then switch to oral therapy after 3–5 years if the patient has a good clinical response. However, there are no data to evaluate the effects of this sequential treatment strategy.

There have been scarce data on the use of migalastat in patients 65 years of age and older, although the drug is not contraindicated in this age group.

### Indications for treatment change—practical issues

Table 3 presents general recommendations regarding FD-specific therapy, including selecting the starting strategy, switching modalities, and therapy withdrawal.

**Table 3:** Recommendations for selection of initial Fabry disease-specific therapy and treatment conversion

Initiate migalastat	Amenable mutation and eGFR > 30 ml/min/1.73 m^2^
	Age 12 years or more and weight for adolescents of at least 45 kg
	Compliance with every-other-day oral drug administration
	No intention by female patients to become pregnant,
	Use of medications that may show drug interactions with ERT affecting its efficacy (e.g., chloroquine, hydroxychloroquine, or amiodarone)
	Patient’s preference
Initiate ERT	All mutation types,
	Age 8 years or more, Chronic kidney disease with
	eGFR < 30 ml/min/1.73 m^2^
	Compliance with intravenous every other week infusion
	Intention by female patients to become pregnant
	Patient’s preference
ERT to migalastat switch	Only amenable mutations,
	Age 12 years or more and weight for adolescents of at least 45 kg
	Use of medications that are not allowed in patients receiving ERT, such as chloroquine, hydroxychloroquine, or amiodarone
	Persistent severe or moderate infusion-associated reactions that do not respond to prophylaxis
	The presence of IgE antibody against agalsidase; may be associated with anaphylaxis
	Anti-drug antibodies presence (against agalsidases), particularly if clinical symptoms worsen
	Patient’s preference
Migalastat to ERT switch	No compliance with every-other-day oral drug administration
	eGFR < 30 ml/min/1.73 m^2^
	Side effects of migalastat therapy such as severe recurrent or persistent headache, dizziness and gastrointestinal symptoms, fatigue, muscle pain, and skin changes
	Women considering pregnancy
	Patient’s preference
Withdrawal of disease-specific therapy (ERT or chaperon)	No response to long-term treatment
	Patient’s request
	Life expectancy less than one year due to severe comorbid illness or due to severe Fabry disease with end-stage heart failure; if not a candidate for heart transplantation
	The permanent severe neurocognitive decline of any cause
	Severe reduction in quality of life and functional status despite disease-specific therapy
	Severe, life-threatening infusion-related adverse reactions despite prophylactic treatment and amenable mutation
	Poor patient adherence to disease-specific therapy (e.g., less than 80% of drug doses taken)

*ERT* Enzyme replacement therapy, *eGFR* Estimated glomerular filtration rate

For patients newly started on any disease-specific therapy, it is essential to ensure that assessments of their cardiac (ECG, 24-h ECG Holter monitor, echocardiogram, cardiac MRI) and kidney status (proteinuria, blood pressure, eGFR) as well as FD-biomarkers (e.g., plasma lyso-Gb3) were performed at baseline and repeated regularly in follow-ups.

In patients currently on well-tolerated and effective ERT, we recommend continuing treatment unless the patient prefers otherwise.

Despite the fact that some studies reported an improvement in pain after initiation of migalastat in previously untreated patients [31], switching from ERT to migalastat may not result in fundamental changes in patient-reported FD-specific pain [9]. Thus, ongoing pain on ERT is not a reason to consider switching modalities.

For patients switching from ERT, migalastat is commonly initiated about 2 weeks after the last dose of ERT based on the infusion interval; however, it may be safely initiated even within days of the previous ERT infusion [27].

Progression of FD may occur in patients previously stable on ERT if their compliance with migalastat is less than on ERT. Therefore, compliance information should be reviewed on a regular visit (for example, by reviewing prescriptions and/or empty medication packages) and by discussions with the patient. In our opinion, patients need to agree that the drug will be discontinued if they do not meet at least 80% of the compliance threshold [40].

Before starting migalastat in naïve patients, they should be informed that a switch to ERT may be recommended if their disease worsens. In patients being considered for a switch from ERT to migalastat or the reverse, it is important to ensure that assessments of their clinical and laboratory state (as listed above) are performed before any treatment switching.

### Pregnancy and lactation

As mentioned above, the patients already receiving migalastat should be advised to stop it before conception and remain off the treatment while breastfeeding. If FD-specific therapy is medically recommended during pregnancy, ERT rather than migalastat must be advised. Several successful pregnancies have been reported in FD women receiving both forms of ERT [41–43].

## Economic considerations of migalastat therapy

In Poland, the monthly costs of migalastat and ERT are similar and covered by the National Health Fund. However, considering the hidden fees related to medical staff involvement, patients arriving at medical facilities, infusion time, and thus wasted working hours, the total costs of migalastat treatment are likely much lower than those of ERT. That is an additional advantage of migalastat use, even if monthly expenses based on drug prices are the same as in our country. However, even within EU due to significant differences in reimbursement healthcare system policies and the expenses incurred directly by patients, the total costs of drugs vary in different countries and that may require additional pharmacoeconomic analysis.

## Conclusions

- Disease-specific therapy should be considered in all FD patients of any age or sex who meet the therapeutic criteria.
- Migalastat is the only oral FD-specific treatment that provides a suitable alternative to intravenous every-other-week recombinant enzyme intravenous infusions in those with amenable genetic variants. Clinical data have provided a growing body of evidence on both the safety and efficacy of migalastat in reducing cardiac hypertrophy and stabilizing kidney function in those with amenable mutations. Long-term follow-up studies demonstrating nearly a decade of treatment experience are underway.
- The ERT conversion to migalastat may be recommended, particularly for those experiencing adverse events during ERT infusions.
- Migalastat can be initiated as the first-line therapy in disease-specific naïve patients who comply with drug use. However, since the data on mutation amenability come from in vitro studies and this medication is relatively new, some caution is needed, e.g. in women considering pregnancy. In carefully selected individual cases, a co-medication of ERT together with migalastat might be considered by the interdisciplinary FD team, although clinical experience with such combined therapy remains insufficient.
- Since migalastat was not directly compared to novel, currently developed treatments, such as plant-origin ERTs and substrate reduction approaches, further clinical trials are needed to assess their superiority.

## Data Availability

All publications that were analysed by the authors to write these recommendations are available in PubMed. The digital object identifiers to locate and download these articles are provided in the reference list.

## References

[1] Germain DP (2010). Fabry disease. Orphanet J Rare Dis.

[2] Nowicki M, Bazan-Socha S, Błażejewska-Hyzorek B, Gellert R, Imiela J, Kaźmierczak J (2020). Enzyme replacement therapy in Fabry disease in Poland: a position statement. Polish Arch Intern Med.

[3] Cairns T, Müntze J, Gernert J, Spingler L, Nordbeck P, Wanner C (2018). Hot topics in Fabry disease. Postgrad Med J.

[4] Choroby nieonkologiczne—Ministerstwo Zdrowia—Portal Gov.pl [Internet]. [cited 2022 Dec 11]. Available from: https://www.gov.pl/web/zdrowie/choroby-nieonkologiczne

[5] van der Veen SJ, Hollak CEM, van Kuilenburg ABP, Langeveld M (2020). Developments in the treatment of Fabry disease. J Inherit Metab Dis.

[6] Galafold|European Medicines Agency [Internet]. [cited 2022 Dec 11]. Available from: https://www.ema.europa.eu/en/medicines/human/EPAR/galafold

[7] Lenders M, Stappers F, Niemietz C, Schmitz B, Boutin M, Ballmaier PJ (2019). Mutation-specific Fabry disease patient-derived cell model to evaluate the amenability to chaperone therapy. J Med Genet.

[8] Modrego A, Amaranto M, Godino A, Mendoza R, Barra JL, Corchero JL (2021). Human α-galactosidase A mutants: priceless tools to develop novel therapies for Fabry disease. Int J Mol Sci.

[9] Hughes DA, Nicholls K, Shankar SP, Sunder-Plassmann G, Koeller D, Nedd K (2017). Oral pharmacological chaperone migalastat compared with enzyme replacement therapy in Fabry disease: 18-month results from the randomised phase III ATTRACT study. J Med Genet.

[10] Benjamin ER, Della Valle MC, Wu X, Katz E, Pruthi F, Bond S (2017). The validation of pharmacogenetics for the identification of Fabry patients to be treated with migalastat. Genet Med.

[11] Ishii S, Chang HH, Kawasaki K, Yasuda K, Wu HL, Garman SC (2007). Mutant alpha-galactosidase A enzymes identified in Fabry disease patients with residual enzyme activity: biochemical characterization and restoration of normal intracellular processing by 1-deoxygalactonojirimycin. Biochem J.

[12] Bichet DG, Torra R, Wallace E, Hughes D, Giugliani R, Skuban N (2021). Long-term follow-up of renal function in patients treated with migalastat for Fabry disease. Mol Genet Metab Rep.

[13] Lenders M, Nordbeck P, Kurschat C, Karabul N, Kaufeld J, Hennermann JB (2020). Treatment of Fabry’s disease with migalastat: outcome from a prospective observational multicenter study (FAMOUS). Clin Pharmacol Ther.

[14] Azevedo O, Gago MF, Miltenberger-Miltenyi G, Sousa N, Cunha D (2020). Fabry disease therapy: state-of-the-art and current challenges. Int J Mol Sci.

[15] Oommen S, Zhou Y, Meiyappan M, Gurevich A, Qiu Y (2019). Inter-assay variability influences migalastat amenability assessments among Fabry disease variants. Mol Genet Metab.

[16] Lenders M, Stappers F, Brand E (2020). In vitro and in vivo amenability to migalastat in Fabry disease. Mol Ther Methods Clin Dev.

[17] Johnson FK, Mudd PN, Bragat A, Adera M, Boudes P (2013). Pharmacokinetics and safety of migalastat HCl and effects on agalsidase activity in healthy volunteers. Clin Pharmacol Drug Dev.

[18] Johnson FK, Mudd PN, Janmohamed SG (2015). Relative bioavailability and the effect of meal type and timing on the pharmacokinetics of migalastat in healthy volunteers. Clin Pharmacol Drug Dev.

[19] Khanna R, Soska R, Lun Y, Feng J, Frascella M, Young B (2010). The pharmacological chaperone 1-deoxygalactonojirimycin reduces tissue globotriaosylceramide levels in a mouse model of Fabry disease. Mol Ther.

[20] Young-Gqamana B, Brignol N, Chang HH, Khanna R, Soska R, Fuller M (2013). Migalastat HCl reduces globotriaosylsphingosine (lyso-Gb3) in Fabry transgenic mice and in the plasma of Fabry patients. PLoS ONE.

[21] Germain DP, Hughes DA, Nicholls K, Bichet DG, Giugliani R, Wilcox WR (2016). Treatment of Fabry’s disease with the pharmacologic chaperone migalastat. N Engl J Med.

[22] Germain DP, Nicholls K, Giugliani R, Bichet DG, Hughes DA, Barisoni LM (2019). Efficacy of the pharmacologic chaperone migalastat in a subset of male patients with the classic phenotype of Fabry disease and migalastat-amenable variants: data from the phase 3 randomized, multicenter, double-blind clinical trial and extension study. Genet Med.

[23] Feldt-Rasmussen U, Hughes D, Sunder-Plassmann G, Shankar S, Nedd K, Olivotto I (2020). Long-term efficacy and safety of migalastat treatment in Fabry disease: 30-month results from the open-label extension of the randomized, phase 3 ATTRACT study. Mol Genet Metab.

[24] Müntze J, Gensler D, Maniuc O, Liu D, Cairns T, Oder D (2019). Oral chaperone therapy migalastat for treating Fabry disease: enzymatic response and serum biomarker changes after 1 year. Clin Pharmacol Ther.

[25] Riccio E, Zanfardino M, Ferreri L, Santoro C, Cocozza S, Capuano I (2020). Switch from enzyme replacement therapy to oral chaperone migalastat for treating Fabry disease: real-life data. Eur J Hum Genet.

[26] Gupta V, Hild SA, Jakkaraj SR, Carlson EJ, Wong HL, Georg GI (2019). N-Butyldeoxygalactonojirimycin induces reversible infertility in male CD rats. Int J Mol Sci.

[27] Hughes DA, Nicholls K, Sunder-Plassmann G, Jovanovic A, Feldt-Rasmussen U, Schiffmann R (2019). Safety of switching to migalastat from enzyme replacement therapy in Fabry disease: experience from the phase 3 ATTRACT study. Am J Med Genet A.

[28] Camporeale A, Bandera F, Pieroni M, Pieruzzi F, Spada M, Bersano A, et al. Effect of migalastat on cArdiac involvement in FabRry disease: MAIORA study. J Med Genet. 2023. https://pubmed.ncbi.nlm.nih.gov/36669872/10.1136/jmg-2022-108768 36669872

[29] Mauer M, Sokolovskiy A, Barth JA, Castelli JP, Williams HN, Benjamin ER (2017). Reduction of podocyte globotriaosylceramide content in adult male patients with Fabry disease with amenable GLA mutations following 6 months of migalastat treatment. J Med Genet.

[30] Lenders M, Nordbeck P, Kurschat C, Eveslage M, Karabul N, Kaufeld J (2022). Treatment of Fabry disease management with migalastat-outcome from a prospective 24 months observational multicenter study (FAMOUS). Eur Hear J Cardiovasc Pharmacother.

[31] Müntze J, Lau K, Cybulla M, Brand E, Cairns T, Lorenz L (2023). Patient reported quality of life and medication adherence in Fabry disease patients treated with migalastat: a prospective, multicenter study. Mol Genet Metab.

[32] Hughes DA, Bichet DG, Giugliani R, Hopkin RJ, Krusinska E, Nicholls K, et al. Long-term multisystemic efficacy of migalastat on Fabry-associated clinical events, including renal, cardiac and cerebrovascular outcomes. J Med Genet. 2022. https://jmg.bmj.com/content/early/2022/12/21/jmg-2022-108669 10.1136/jmg-2022-108669 PMC10359570 36543533

[33] Ortiz A, Germain DP, Desnick RJ, Politei J, Mauer M, Burlina A (2018). Fabry disease revisited: management and treatment recommendations for adult patients. Mol Genet Metab.

[34] Goods Administration T. Australian public assessment report for migalastat about the therapeutic goods administration (TGA). 2018. Available from: https://www.tga.gov.au

[35] List of Pregnancy Exposure Registries|FDA [Internet]. [cited 2022 Dec 11]. Available from: https://www.fda.gov/science-research/womens-health-research/list-pregnancy-exposure-registries

[36] Wanner C, Arad M, Baron R, Burlina A, Elliott PM, Feldt-Rasmussen U (2018). European expert consensus statement on therapeutic goals in Fabry disease. Mol Genet Metab.

[37] Chimenti C, Nencini P, Pieruzzi F, Feriozzi S, Mignani R, Pieroni M (2020). The GALA project: practical recommendations for the use of migalastat in clinical practice on the basis of a structured survey among Italian experts. Orphanet J Rare Dis.

[38] Bichet DG, Aerts JM, Auray-Blais C, Maruyama H, Mehta AB, Skuban N (2021). Assessment of plasma lyso-Gb3 for clinical monitoring of treatment response in migalastat-treated patients with Fabry disease. Genet Med.

[39] Sirrs S, Bichet DG, Iwanochko RM, Khan A, Moore D, Oudit G, et al. 2017 Canadian Fabry disease guidelines; 2017.

[40] Baumgartner PC, Haynes RB, Hersberger KE, Arnet I (2018). A systematic review of medication adherence thresholds dependent of clinical outcomes. Front Pharmacol.

[41] Wendt S, Whybra C, Kampmann C, Teichmann E, Beck M (2005). Successful pregnancy outcome in a patient with Fabry disease receiving enzyme replacement therapy with agalsidase alfa. J Inherit Metab Dis.

[42] Politei JM (2010). Treatment with agalsidase beta during pregnancy in Fabry disease. J Obstet Gynaecol Res.

[43] Germain DP, Bruneval P, Tran TC, Balouet P, Richalet B, Benistan K (2010). Uneventful pregnancy outcome after enzyme replacement therapy with agalsidase beta in a heterozygous female with Fabry disease: a case report. Eur J Med Genet.

