# Correction: A review and recommendations for oral chaperone therapy in adult patients with Fabry disease

**DOI:** 10.1186/s13023-024-03140-x

**Published:** 2024-03-28

**Authors:** Michał Nowicki, Stanisława Bazan‑Socha, Beata Błażejewska‑Hyżorek, Mariusz M. Kłopotowski, Monika Komar, Mariusz A. Kusztal, Tomasz Liberek, Jolanta Małyszko, Katarzyna Mizia‑Stec, Zofia Oko‑Sarnowska, Krzysztof Pawlaczyk, Piotr Podolec, Jarosław Sławek, the Polish Fabry Disease Collaborative Group

**Affiliations:** 1 Department of Nephrology, Hypertension and Kidney Transplantation, Central University Hospital, Medical University of Lodz, Pomorska 251, 92‑213 Lodz, Poland; 2 Department of Internal Medicine, Faculty of Medicine, Jagiellonian University Medical College, Krakow, Poland; 3 2Nd Department of Neurology, Institute of Psychiatry and Neurology, Warsaw, Poland; 4 Department of Interventional Cardiology and Angiology, Cardinal Wyszynski National Institute of Cardiology-National Research Institute, Warsaw, Poland; 5 Department of Cardiac and Vascular Diseases, Institute of Cardiology, Jagiellonian University Medical College, Krakow, Poland; 6 Department of Nephrology and Transplantation Medicine, Wroclaw Medical University, Wroclaw, Poland; 7 Department of Nephrology, Transplantology and Internal Medicine, Medical University of Gdańsk, Gdańsk, Poland; 8 Department of Nephrology, Dialysis and Internal Medicine, Medical University of Warsaw, Warsaw, Poland; 9 First Department of Cardiology, Faculty of Medicine in Katowice, Medical University of Silesia, Katowice, Poland; 10 1St Department of Cardiology, Poznan University of Medical Sciences, Poznan, Poland; 11 Department of Nephrology, Transplantology and Internal Medicine, Poznań University of Medical Sciences, Poznan, Poland; 12 Department of Neurological-Psychiatric Nursing, Department of Neurology and Stroke, Faculty of Health Sciences, St. Adalbert Hospital, Medical University of Gdansk, Gdańsk, Poland

**Correction** **:** **Orphanet J Rare Dis (2024) 19:16**

**https://doi.org/10.1186/s13023-024-03028-w**

Following publication of the original article [1], we have been notified that there was a mistake in the body text of the article.

It is now:

**Polish experience with migalastat therapy**

At the end of December 2024, 32 FD patients are treated with migalastat in Poland, and none was discontinued.

It should be:

**Polish experience with migalastat therapy**

At the end of December 2023, 32 FD patients are treated with migalastat in Poland, and none was discontinued.
